# Spatial regulation of the polarity kinase PAR-1 by parallel inhibitory mechanisms

**DOI:** 10.1242/dev.171116

**Published:** 2019-03-25

**Authors:** Andrew W. Folkmann, Geraldine Seydoux

**Affiliations:** Department of Molecular Biology and Genetics, HHMI, Johns Hopkins University, School of Medicine, 725 N. Wolfe Street, Baltimore, MD 21205, USA

**Keywords:** Polarity, Kinase, PAR proteins, MEX-6, P granules, PAR-3

## Abstract

The MARK/PAR-1 family of kinases are conserved regulators of cell polarity that share a conserved C-terminal kinase-associated domain (KA1). Localization of MARK/PAR-1 kinases to specific regions of the cell cortex is a hallmark of polarized cells. In *Caenorhabditis* *elegans* zygotes, PAR-1 localizes to the posterior cortex under the influence of another polarity kinase, aPKC/PKC-3. Here, we report that asymmetric localization of PAR-1 protein is not essential, and that PAR-1 kinase activity is regulated spatially. We find that, as in human MARK1, the PAR-1 KA1 domain is an auto-inhibitory domain that suppresses kinase activity. Auto-inhibition by the KA1 domain functions in parallel with phosphorylation by PKC-3 to suppress PAR-1 activity in the anterior cytoplasm. The KA1 domain also plays an additional role that is essential for germ plasm maintenance and fertility. Our findings suggest that modular regulation of kinase activity by redundant inhibitory inputs contributes to robust symmetry breaking by MARK/PAR-1 kinases in diverse cell types.

## Abstract (precis)

**Highlighted Article:** Auto-inhibition and regulation by aPKC/PKC-3 function in parallel to restrict the kinase activity of the cell polarity regulator PAR-1 to the posterior cytoplasm of *C. elegans* zygotes.

## INTRODUCTION

Cell polarity is central to many cellular and developmental processes, such as axon specification, cell migration, tissue morphogenesis and asymmetric cell division. The kinase PAR-1 is a conserved regulator of cell polarity in eukaryotes. In polarized cells, PAR-1 often localizes to specific membrane domains. For example, in *Drosophila* oocytes and *Caenorhabditis* *elegans* zygotes, PAR-1 localizes to the posterior cell cortex, and this asymmetry is essential to concentrate germ cell fate determinants to the posterior end of the embryo at which the germline will form (Goldstein and Macara, 2007; Guo and Kemphues, 1995; Shulman et al., 2000; Tomancak et al., 2000; Wu and Griffin, 2017). The mechanisms that regulate PAR-1 activity in space are central to the polarization process, but are still poorly understood. For example, although asymmetric localization of PAR-1 at the cell cortex is a prominent characteristic of polarized cells, studies in *C. elegans* have suggested that cortical localization is not essential for PAR-1 function (Boyd et al., 1996) and that asymmetric PAR-1 activity in the cytoplasm is crucial for zygote polarity (Griffin et al., 2011). PAR-1 has also been reported to interact with non-muscle myosin, but the significance of these interactions remains unclear (Guo and Kemphues, 1996).

**Fig. 1.**
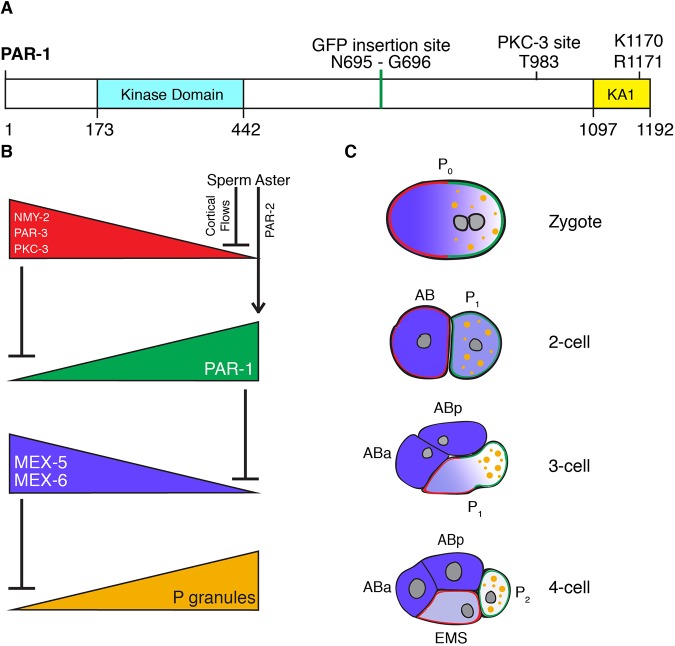
**PAR-1 and polarization of the *C. elegans* embryo.** (A) Schematic of *C. elegans* PAR1 (PAR-1a isoform as predicted by WormBase WS265). The kinase domain and KA1 domain are separated by a long linker sequence. The GFP insertion site, phosphorylation site for PKC-3 and two conserved basic residues in the KA1 domain are indicated. (B) Genetic hierarchy that governs polarization of the zygote. In this and all other figures, anterior is to left and posterior is to the right. The sperm aster induces NMY-2 powered cortical flows that transport PKC-3 and PAR-3 towards the anterior. The sperm aster microtubules also recruit PAR-2 to the cortex nearest the sperm aster. PKC-3 excludes PAR-1 from the anterior and PAR-2 recruits PAR-1 to the posterior. PAR-1 phosphorylates MEX-6 and its homolog MEX-5 in the posterior cytoplasm causing a local increase in mobility that leads to accumulation of MEX-6 and MEX-5 in the anterior cytoplasm. MEX-6 and MEX-5 together oppose P granule assembly. (C) Diagrams of embryos undergoing the first two divisions. Color scheme follows B. AB_a_ and AB_p_ are the daughters of AB. EMS and P_2_ are the daughters of P_1_.

PAR-1 belongs to the MARK/PAR-1 family of kinases that shares a conserved organization: an N-terminal kinase domain followed by a linker region and a C-terminal kinase-associated (KA1) domain (Fig. 1A) (Marx et al., 2010). The linker region contains a phosphorylation site for atypical protein kinase C (aPKC; the aPKC homolog is denoted as PKC-3) (Hurov et al., 2004). In *Drosophila* and *C. elegans*, aPKC localizes in an anterior domain that is complementary to the PAR-1 domain, and phosphorylation of PAR-1 by aPKC prevents PAR-1 from associating with the aPKC cortical domain (Doerflinger et al., 2010; Motegi et al., 2011; Ramanujam et al., 2018). Experiments with the human PAR-1 homolog Par1b (MARK2) have also suggested that phosphorylation by aPKC inhibits kinase activity (Hurov et al., 2004). The kinase activity of MARK/PAR-1 family members is also regulated via an intramolecular auto-inhibition mechanism. The KA1 domain binds to the kinase catalytic domain and inhibits kinase activity (Emptage et al., 2017, 2018). The KA1 domain contains a basic surface that binds to anionic phospholipids *in vitro* and targets PAR-1 to membranes *in vivo* (Goransson et al., 2006; Moravcevic et al., 2010; Motegi et al., 2011; Ramanujam et al., 2018). The basic amino acids that are required for binding to phospholipids are also required for auto-inhibition, and recruitment of the KA1 domain to artificial membranes is sufficient to relieve kinase auto-inhibition *in vitro* (Emptage et al., 2017). Recruitment to a membrane can also be enhanced by membrane proteins that bind to regions that are adjacent to the KA1 domain or to the KA1 domain itself (Moravcevic et al., 2010; Ramanujam et al., 2018). Together, these observations suggest that binding of PAR-1 to membrane proteins coincidently localizes and activates PAR-1 kinase activity (Emptage et al., 2017, 2018; Moravcevic et al., 2010). How regulation by the KA1 domain coordinates with regulation by PKC-3 to restrict PAR-1 activity in space is not yet known and is the subject of this study.

In this study, we examined PAR-1 regulation in the zygote of the nematode *C. elegans*. This is a well-studied example of a polarized cell (Lang and Munro, 2017) (Fig. 1B,C). Under the influence of the sperm aster, the zygote becomes polarized along its long anterior-posterior axis during the first cell cycle (Fig. 1B,C). The sperm aster triggers a cortical flow of actomyosin that enriches the scaffold PAR-3 and its partner, PKC-3, in the anterior cortex of the zygote (Munro et al., 2004; Rodriguez et al., 2017). The sperm aster also recruits the polarity regulator PAR-2 to the cortex that is nearest the aster (posterior). Phosphorylation by PKC-3 excludes PAR-1 from the anterior cortex and cytoplasm, and binding to PAR-2 enriches PAR-1 on the posterior cortex (Motegi et al., 2011; Ramanujam et al., 2018). Posteriorly enriched PAR-1 in turn phosphorylates PAR-3, which excludes it from the posterior cortex (Motegi et al., 2011; Sailer et al., 2015). Asymmetric PAR-1 also drives the asymmetric distribution of MEX-5, MEX-6 and P granules in the cytoplasm in zygotes. MEX-5 and MEX-6 are redundant RNA-binding proteins that are phosphorylated by PAR-1 (Griffin et al., 2011; Schubert et al., 2000; Tenlen et al., 2008). Phosphorylation causes MEX-5/6 to increase their diffusion in the posterior cytoplasm and redistribute in an anterior-rich gradient opposite PAR-1 (Griffin et al., 2011; Schubert et al., 2000; Tenlen et al., 2008). MEX-5/6 in turn pattern the P granules (Fig. 1B). P granules are RNA-protein condensates that are specific to the germline (Seydoux, 2018). P granule assembly in embryos depends on MEG-3 and MEG-4; two intrinsically disordered proteins that phase separate with RNA and recruit other P granule components (Wang et al., 2014). MEX-5/6 promote the dissolution of MEG-3/4 condensates in the anterior cytoplasm, causing MEG-3/4 and other P granule components to assemble only in the posterior (Smith et al., 2016). In mutants that lack *par-1* activity, the MEX-5/6 gradient does not form and P granules disassemble throughout the zygote. In contrast, in mutants that lack *mex-5* and *mex-6*, P granules assemble throughout the zygote (Gallo et al., 2010; Schubert et al., 2000). These observations predict that uniform PAR-1 would also lead to uniform assembly of P granules in zygotes.

PAR-1, MEX-5/6 and P granule asymmetries persist during the first cleavage to yield distinct daughter cells: the anterior somatic blastomere (AB), which inherits high levels of MEX-5/6, and the posterior germline blastomere (P_1_), which inherits high levels of PAR-1 and P granules (Fig. 1C). In the next cell cycle, AB divides symmetrically, and P_1_ divides asymmetrically to generate another pair of somatic and P blastomeres. Asymmetric segregation of PAR-1, MEX-5/6 and P granules is repeated in each P blastomere until the birth of the germline founder cell P_4_ (Guo and Kemphues, 1995; Schubert et al., 2000; Strome and Wood, 1983).

To understand how regulation by PKC-3 and the KA1 domain impact PAR-1 activity *in vivo*, we modified the *par-1* locus using genome editing and examined the effect on the distributions of PAR-1, PAR-3, MEX-5, MEX-6 and MEG-3 in live embryos. Strikingly, we found that neither regulation by PKC-3, nor regulation by the KA1 domain, is essential to polarize PAR-1 activity. Coordinate regulation by both mechanisms, however, is needed for robust cell polarization. Our results suggest that PKC-3 and the KA1-domain function in parallel to create reinforcing PAR-1 activity gradients.

## RESULTS

### Tagging of *par-1* locus with GFP reveals nearly ubiquitous expression

To visualize the distribution of PAR-1 protein *in vivo*, we tagged the *par-1* locus with GFP using CRISPR genome editing (Paix et al., 2015). The *par-1* locus is predicted to produce several isoforms with unique N-terminal exons and a common C-terminal exon that encodes the KA1 domain (WormBase WB266). We first attempted to tag all isoforms by introducing mEGFP immediately upstream of the stop codon in the C-terminal exon. We recovered several animals that were heterozygous for the edit, but none was fertile (data not shown). As an alternative approach, we integrated mEGFP at amino acid 695, near the center of the ‘linker’ region of PAR-1 (Fig. 1A), a domain that is also present in all PAR-1 isoforms. We obtained several fertile *par-1::GFP* edits, and derived a homozygous strain that was fully viable and fertile (JH3616; Table S1).

**Fig. 2.**
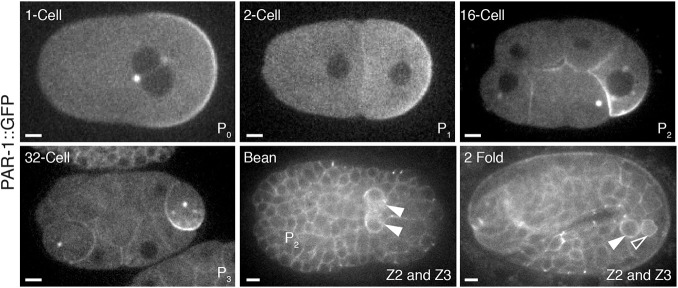
**PAR-1::GFP is expressed ubiquitously during embryogenesis and is enriched in the embryonic germline.** Photomicrographs of live embryos expressing PAR-1::GFP. PAR-1::GFP is enriched at cell cortices and segregated preferentially to the germline. In each panel, the embryonic stage is indicated in the upper left and the names of the germline blastomere for that stage is indicated in the lower right. Solid arrowheads indicate the two primordial germ cells (Z2 and Z3). A smaller open arrowhead in the 2-fold stage panel points to the lobe of one of the primordial germ cells (Abdu et al., 2016). Scale bars: 5 μm.

Consistent with previous reports in zygotes (Griffin et al., 2011; Guo and Kemphues, 1995), PAR-1::GFP was enriched on the posterior cortex, in a weak gradient in the posterior cytoplasm, on centrosomes and weakly on P granules (Fig. 2). Asymmetric segregation was repeated in each P blastomere, with PAR-1::GFP enriched on the cortex that was destined for the next P cell daughter (Fig. 2). Enrichment of PAR-1 in the germ lineage persisted through gastrulation. Cortical PAR-1 could be detected in most, and perhaps all, embryonic cells, but was present at higher levels in the primordial germ cells Z2 and Z3, which are daughters of the P_4_ blastomere (Fig. 2).

### Threonine 983 is required for PAR-1 protein asymmetry and embryonic viability

**Fig. 3.**
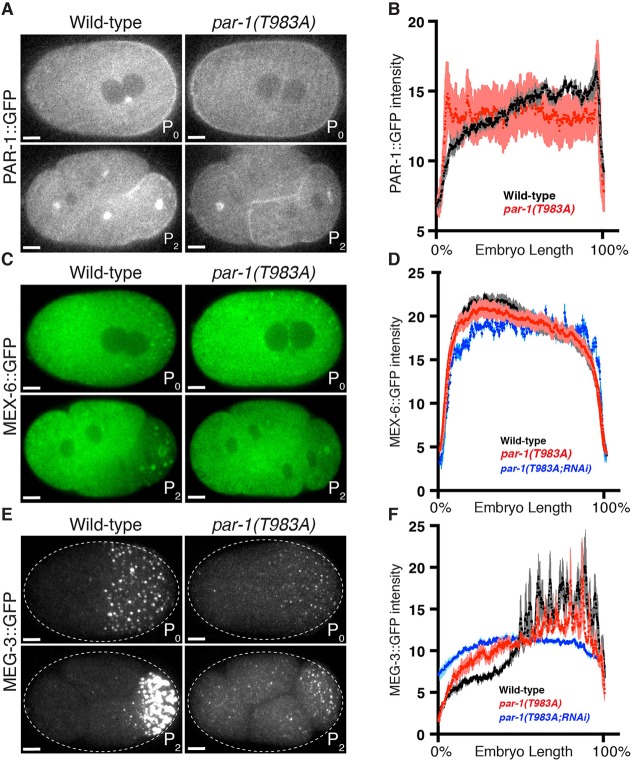
**Threonine 983 contributes to, but is not essential for, polarized PAR-1 activity.** (A,C,E) Photomicrographs of live wild-type and *par-1(T983A)* zygotes and four-cell embryos with GFP-tagged PAR-1 (A), MEX-6 (C) or MEG-3 (E). Although PAR-1::GFP does not enrich in the posterior in *par-1(T983A)*, GFP::MEX-6 and GFP::MEG-3 still segregate asymmetrically. (B,D,F) Graphs plotting the relative signal intensity of the indicated GFP fusions in zygotes of the indicated genotypes. Four zygotes at pronuclear meeting were analyzed for each condition. Error bars represent s.e.m. MEX-6 and MEG-3 gradients in *par-1(T983A)* are shallower than in the wild type. *par-1(T983A; RNAi)* embryos were depleted of PAR-1(T983A) by *par-1* RNAi and exhibit no gradients, which confirms that PAR-1(T983A) is required for the observed gradients. Scale bars: 5 μm.

Asymmetric localization of PAR-1 in zygotes depends on the anterior-enriched kinase PKC-3, which phosphorylates PAR-1 on threonine 983 (Hurov et al., 2004; Motegi et al., 2011) (Fig. 1). Previous studies that used *par-1* transgenes showed that replacement of threonine 983 with alanine was sufficient to eliminate PAR-1 asymmetry (Motegi et al., 2011; Ramanujam et al., 2018). To confirm these results at the *par-1* locus, we introduced the T983A mutation using genome editing in the PAR-1::GFP line (Fig. 3A; Materials and Methods). PAR-1 is provided maternally to embryos, therefore we examined embryos that were derived from mothers homozygous for the edit. As expected, we found that PAR-1::GFP(T983A) was no longer excluded from the anterior cortex and anterior cytoplasm, and appeared to be uniformly distributed throughout the cytoplasm (Fig. 3A). Quantitation of GFP fluorescence in zygotes confirmed that PAR-1(T983A)::GFP is expressed at a level similar to wild-type PAR-1::GFP (Fig. S1), but no longer forms a posterior-rich gradient (Fig. 3A). PAR-1(T983A)::GFP segregated equally to AB and P_1_ blastomeres at the first cleavage, and continued to segregate equally in subsequent divisions (Fig. 3A and data not shown). In wild-type embryos, AB divides before P_1_ (Tavernier et al., 2015). We observed that in *par-1(T983A)::GFP* embryos, the AB and P_1_ blastomeres divided synchronously and their progeny also divided synchronously (Fig. S2A). Consistent with these embryos undergoing synchronous divisions, nearly all *par-1(T983A)* embryos (98%) did not survive embryogenesis (JH3614; Table S1). The small percentage of embryos that survived grew into sterile adults (JH3614; Table S1). We conclude that the PKC phosphorylation site T983 is essential for PAR-1 asymmetry, cell cycle asynchrony and embryonic viability.

### Threonine 983 contributes but is not essential for polarized PAR-1 activity

To examine the consequences of symmetric PAR-1 on zygote polarity in more detail, we introduced the T983A mutation in the untagged *par-1* locus in strains that had previously been edited with fluorescent tags at the *mex-6* and *meg-3* loci (JH3623 and JH3614; Table S2). We also generated a strain that co-expressed PAR-1(T983A)::GFP and mCherry::MEX-5. We found that mCherry::MEX-5, MEX-6::GFP and MEG-3::GFP still localized asymmetrically in zygotes and in the P_1_ blastomere in *par-1(T983A)* embryos (embryos derived from homozygous *par-1(T983A)* mothers; Fig. 3B-F and Fig. S2B). The MEX-5, MEX-6 and MEG-3 gradients were not as robust as in the wild type (Fig. 3E,F and Fig. S2C) and weakened further during mitosis (Fig. S2D-E and Fig. S3A). To test whether P granule asymmetry was still under the control of *par-1* and *mex-5/6*, we used RNA-mediated interference (RNAi) to deplete *par-1* and *mex-5/6* in *par-1(T983A)* zygotes that expressed MEG-3::GFP. We found that, as in the wild type, depletion of PAR-1 protein using *par-1* RNAi caused dissolution of P granules throughout the cytoplasm, which is consistent with a requirement for PAR-1 activity for P granule asymmetry. As expected, RNAi against *mex-5* and *mex-6* had the opposite effect, causing P granules to assemble throughout the cytoplasm (Fig. S3B,C) (Gallo et al., 2010). Interestingly, RNAi against MEX-6 alone restored asymmetric P granules during mitosis in *par-1(T983A)* zygotes (Fig. S3A), which suggests that lowering the dosage of MEX-5/6 activity allows for more robust asymmetric patterning by the *par-1(T983A)* allele. These observations confirm that, as seen with wild-type PAR-1, PAR-1(T983A) polarizes P granules by opposing the P granule-disassembling activity of MEX-5/6. PAR-1(T983A), however, appears to be less active than wild-type PAR-1, perhaps because PAR-1(T983A) is not enriched in the posterior.

### The KA1 domain downregulates PAR-1 kinase activity *in vitro*

**Fig. 4.**
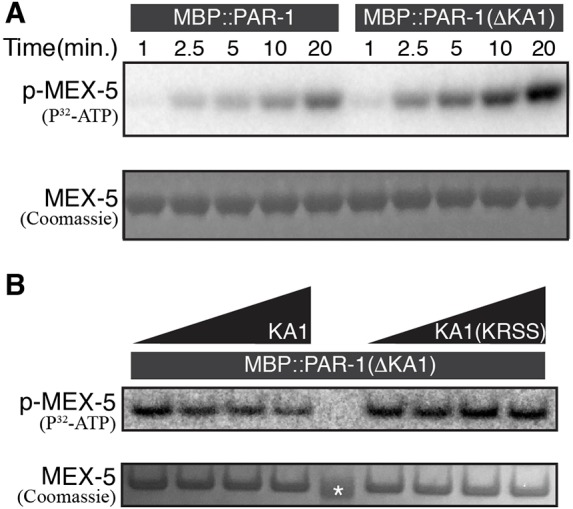
**The KA1 domain inhibits PAR-1 kinase activity *in vitro*.** (A) Autoradiograph of a time course kinase assay using recombinant MBP::PAR-1 or MBP::PAR-1(ΔKA1) kinase, and MBP::MEX-5(452-460) as a substrate. Reactions were performed in the presence of P^32^-ATP for the times indicated. Bottom panel shows Coomassie Blue staining to control for loading of MEX-5. The experiment was repeated three times; a representative gel is shown. For loading control of kinase refer to full gels shown in Fig. S4C. (B) Autoradiograph of a kinase assay using recombinant MBP::PAR-1(ΔKA1) kinase and titrating in His(6)::KA1 or His(6)::KA1(KRSS) at 0, 5 mM, 20 mM and 50 mM concentrations (left to right). Reactions were performed in the presence of P^32^-ATP for 10 min. The experiment was repeated three times; a representative gel is shown. Bottom panel shows Coomassie Blue staining to control for loading of MEX-5. Asterix indicates 37 kDa molecular mass marker. For loading control of kinase refer to full gel shown in Fig. S4D.

The observation that *par-1(T983A*) zygotes retain some polarity raises the possibility that another mechanism besides regulation by PKC-3 confines PAR-1 activity to the posterior. The KA1 domain has been shown to function as an auto-inhibitory domain in human MARK1 kinases (Emptage et al., 2017, 2018). To test whether the KA1 domain of *C. elegans* PAR-1 has a similar regulatory activity, we first used an *in vitro* assay for PAR-1 kinase activity that uses MEX-5 as a substrate (Fig. 4) (Griffin et al., 2011). We tested recombinant full-length PAR-1, PAR-1(ΔKA1) that lacked the KA1 domain, and the KA1 domain itself (Fig. S4A,B). We found MBP::PAR-1(ΔKA1) has a higher basal kinase activity (∼1.5×) compared with MBP::PAR-1 (Fig. 4A and Fig. S4C). Addition of HIS::KA1 in trans was sufficient to reduce the kinase activity of MBP::PAR-1(ΔKA1) (Fig. 4B and Fig. S4D). In human MARK1, mutations in amino acids K773 to R774 eliminate the inhibitory activity of the KA1 domain (Emptage et al., 2017). These amino acids are conserved in *C. elegans* PAR-1 (K1170 to R1171; Fig. 1A). A KA1 domain that contained mutations at these two sites (K1170S R1171S, abbreviated to KRSS) was no longer able to reduce MBP::PAR-1(ΔKA1) kinase activity (Fig. 4B and Fig. S4D). These data confirm that the KA1 domain of *C. elegans* PAR-1 functions as a kinase auto-inhibitory domain.

### The KA1 domain contributes to but is not essential for polarized PAR-1 activity

**Fig. 5.**
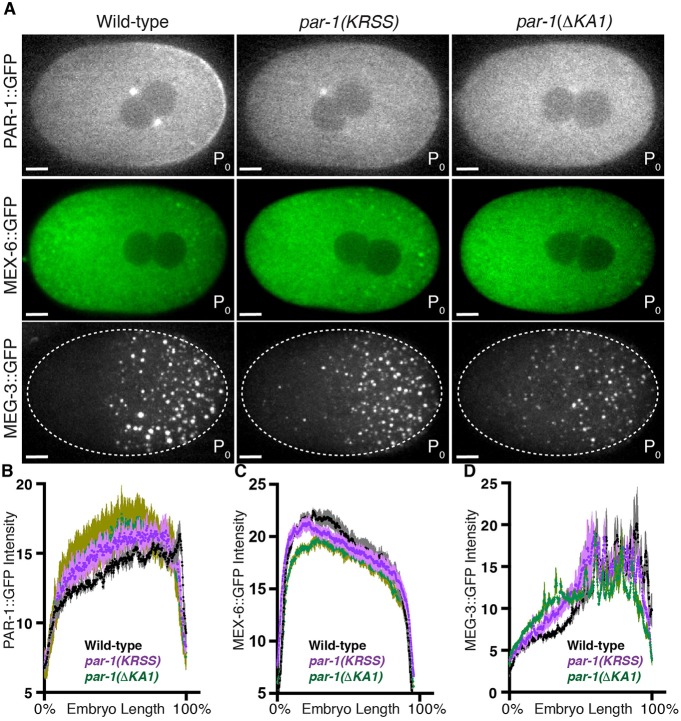
**The KA1 domain contributes to, but is not essential for, polarized PAR-1 activity.** (A) Photomicrographs of live wild-type, *par-1(KRSS)* or *par-1(ΔKA1)* zygotes with GFP-tagged PAR-1, MEX-6 or MEG-3. (B-D) Graphs plotting the relative signal intensity of the indicated GFP fusions in zygotes of the indicated genotypes. Four zygotes at pronuclear meeting were analyzed for each condition. Error bars represent s.e.m. The reference wild-type data are also plotted in Fig. 3D,F. Scale bars: 5 μm.

**Fig. 6.**
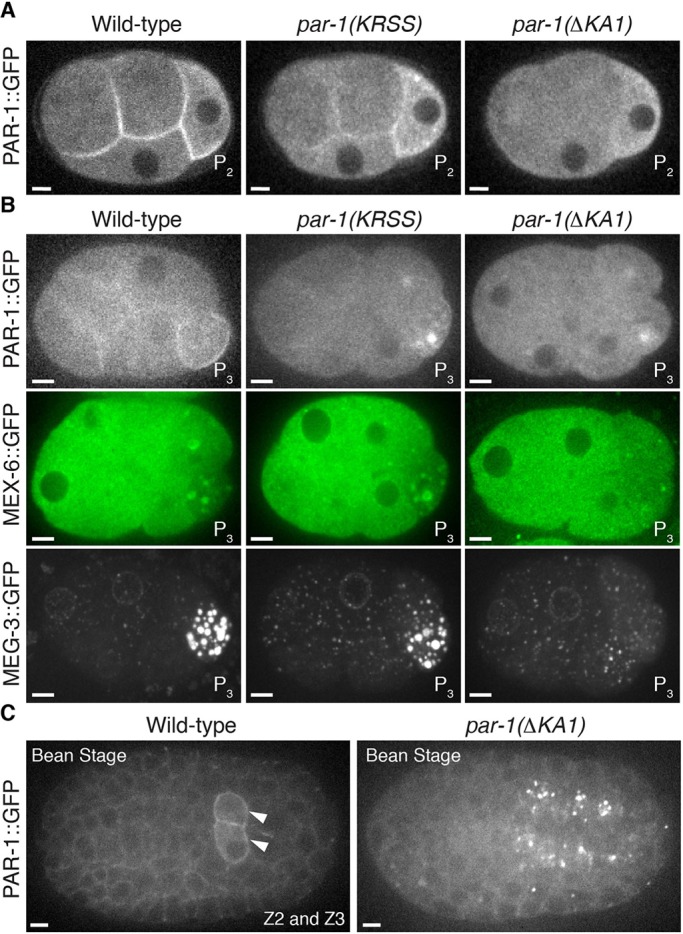
**The KA1 domain is required for efficient asymmetric segregation of PAR-1 to the P lineage.** (A) Photomicrographs of live wild-type, *par-1(KRSS)* or *par-1(ΔKA1)* four-cell embryos with GFP-tagged PAR-1. Note that PAR-1(ΔKA1) does not enrich on cell cortices but still segregates preferentially to P_2_. (B) Photomicrographs of live wild-type, *par-1(KRSS)* or *par-1(ΔKA1)* eight-cell embryos with GFP-tagged PAR-1, MEX-6 or MEG-3. PAR-1 and P granules do not segregate efficiently to P_3_ in *par-1(ΔKA1)* embryos. (C) Photomicrographs of live wild-type and *par-1(ΔKA1)* bean-stage embryos expressing GFP-tagged PAR-1. PAR-1 is enriched in the primordial germ cells (arrowheads) in the wild type, but not in *par-1(ΔKA1)*. The identity of the bright foci of PAR-1::GFP(ΔKA1) is not known. Scale bars: 5 μm.

To examine the role of the KA1 domain *in vivo*, we introduced the KRSS mutations or deleted the KA1 domain (abbreviated to ΔKA1) using genome editing in the PAR-1::GFP, MEX-6::GFP and MEG-3::GFP strains. The KA1 domain, including the conserved amino acids K1170 and R1171, has been proposed to mediate electrostatic interactions with acidic phospholipids to help target MARK kinases to cell membranes (Moravcevic et al., 2010; Ramanujam et al., 2018). Mutations in the KA1 domain in the context of a *par-1* transgene were reported to disrupt PAR-1 localization to membranes (Ramanujam et al., 2018). Consistent with these observations, the cortical localization of PAR-1(KRSS)::GFP and PAR-1(ΔKA1)::GFP was greatly reduced; PAR-1(ΔKA1)::GFP showed the greatest reduction, with no detectable membrane enrichment at any stage (Figs 5A and 6). PAR-1(KRSS)::GFP and PAR-1(ΔKA1)::GFP still formed a posterior-rich gradient across the zygote cytoplasm and localized to centrosomes, as has been observed for wild-type PAR-1. *par-1(KRSS)* and *par-1(ΔKA1)* zygotes polarized MEX-6::GFP and MEG-3::GFP almost as robustly as the wild type, although small MEG-3 granules were present in the anterior cytoplasm (Fig. 5). These observations suggest that PAR-1 activity is not as efficiently suppressed in the anterior cytoplasm in the absence of the KA1 domain. We conclude that the KA1 domain contributes to, but is not essential for, suppression of PAR-1 activity in the anterior.

In addition to its primary role in localizing factors in the cytoplasm, PAR-1 also plays a supporting role in excluding anterior PAR proteins, in particular PAR-3, from the posterior cortex (Sailer et al., 2015). Depletion of PAR-1 by RNAi results in a weak, but measurable, increase in the number of PAR-3 cortical clusters in the posterior domain in zygotes at the metaphase stage (Fig. S5; Sailer et al., 2015). We found that *par-1(ΔKA1*) and *par-1(K1170S R1170S)* excluded PAR-3 cortical clusters from the posterior domain as efficiently as the wild type (Fig. S5). Furthermore, ectopically localized cortical PAR-1(T983A) also excluded PAR-3 clusters from the posterior cortex and was not sufficient to displace PAR-3 from the anterior cortex (Fig. S5). Taken together, these data indicate that PAR-1 cortical enrichment or asymmetry is neither necessary nor sufficient to pattern PAR-3 at the cortex in the zygote.

### The KA1 domain is required for efficient asymmetric segregation of PAR-1 in the P lineage and for fertility

After the one-cell stage, *par-1(KRSS)* embryos maintained PAR-1, MEX-6 and MEG-3 asymmetry during each P blastomere division (Fig. 6) and *par-1(KRSS)* embryos grew up into fertile adults (JH3612; Table S1). *par-1(KRSS)* lines could be propagated in the homozygous state for several generations with no apparent abnormalities (Table S1). In contrast, *par-1(ΔKA1)* embryos did not segregate PAR-1, MEX-6 and MEG-3 asymmetrically after the four-cell stage. GFP::PAR-1 is enriched in germline blastomeres and primordial germ cells, but no such enrichment was observed in embryos that expressed GFP::PAR-1(ΔKA1) (Fig. 6). *par-1(ΔKA1)* embryos were viable but developed into sterile adults (maternal effect sterility), and the *par-1(ΔKA1)* lines could only be propagated as balanced heterozygous strains (JH3613; Table S1). We conclude that the KA1 domain is not essential for polarization of zygotes or for viability, but is required for polarization of P blastomeres and for fertility.

### The KA1 domain is required for polarized activity of PAR-1(T983A)

**Fig. 7.**
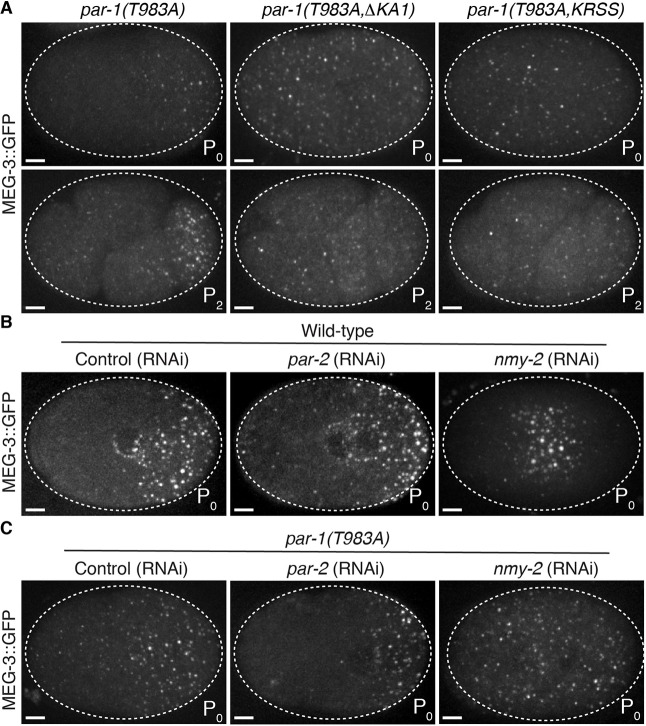
**PKC-3 functions redundantly with KA1 and NMY-2 to inhibit PAR-1 activity in the anterior.** (A) Photomicrographs of live *par-1(T983A)*, *par-1(T983A KRSS)* or *par-1(T983A ΔKA1)* zygotes (top row) and four-cell embryos (bottom row) with GFP-tagged MEG-3. In *par-1(T983A KRSS)* or *par-1(T983A ΔKA1)* embryos, P granules assemble throughout the cytoplasm and are not asymmetrically segregated. (B,C) Photomicrographs of GFP-tagged MEG-3 in live wild-type (B) or *par-1(T983A)* (C) zygotes depleted for *par-2* or *nmy-2* by RNAi. Depletion of *nmy-2*, but not *par-2*, eliminates P granule asymmetry. In *nmy-2* (RNAi), PKC-3 is enriched at the cortex throughout the zygote (Rodriguez et al., 2017) and thus inhibits P granule assembly at the periphery, unless T983 is mutated. Scale bars: 5 μm.

To test whether regulation by the KA1 domain becomes essential in zygotes in the absence of regulation by PKC-3, we mutated K1170 and R1171 to serine, or deleted the KA1 domain, in the *par-1(T983A)* allele. We found that hermaphrodites that were heterozygous for *par-1(T983A KRSS)* or *par-1(T983A ΔKA1)* generated progeny that were mostly all sterile (Table S1). This dominant-maternal effect sterility made it impossible to maintain strains with these combinations of edits for more than one or two generations, and therefore we were not able to characterize these mutants in all backgrounds. We were able to recover and characterize the embryos of 16 fertile *par-1(T983A KRSS)* and *par-1(T983A ΔKA1)* homozygous hermaphrodites in the MEG-3::GFP background. We found that in all zygotes examined (*n*=17), MEG-3::GFP was uniformly distributed and formed granules throughout the zygote cytoplasm (Fig. 7A). This phenotype contrasts with the phenotypes of the T983A mutant and KA1 mutants, ‘single’ mutants, in which P granules were restricted to the posterior (Figs 3E, 5A, 7A). A uniform distribution of P granules is also seen in zygotes that have been depleted of *mex-5* and *mex-6*, and is the opposite phenotype of *par-1(RNAi)* zygotes, in which no P granules form (Fig. S3B). We conclude that, in the absence of regulation by both aPKC and KA1, PAR-1 activity is constitutively high throughout the zygote cytoplasm.

### Cortical flows are required to promote auto-inhibition by the KA1 domain in the anterior cytoplasm

It has been proposed that auto-inhibition by the KA1 domain is regulated by membrane-associated proteins that recruit PAR-1 to the membrane and facilitate binding of the KA1 domain to phospholipids, thus relieving auto-inhibition (Emptage et al., 2017, 2018; Moravcevic et al., 2010). PAR-1 is known to bind to two membrane-associated proteins in zygotes: PAR-2, a polarity regulator that is enriched in the posterior cortex, and NMY-2, a myosin that is enriched in the anterior cortex (Boyd et al., 1996; Guo and Kemphues, 1996; Motegi et al., 2011; Ramanujam et al., 2018). To test which might be required for polarization in the absence of regulation by PKC-3 we inactivated each using RNAi in *par-1(T983A)* zygotes. We found that inactivation of PAR-2 by RNAi did not prevent P granule asymmetry (Fig. 7B,C), which is consistent with previous reports that PAR-2 is not essential for polarization of PAR-1 activity (Boyd et al., 1996; Griffin et al., 2011; Ramanujam et al., 2018). In contrast, inactivation of *nmy-2* led to uniform P granules (Fig. 7C), as seen in *par-1(T983A KRSS)* and *par-1(T983A ΔKA1)* (Fig. 7A). This observation suggests that NMY-2, or a factor that is regulated by NMY-2, is required to pattern auto-inhibition by the KA1 domain.

During cortical flows, NMY-2 becomes enriched in the anterior cortex. Remarkably, we found that during flows PAR-1(T983A)::GFP also becomes enriched in the anterior cortex in a non-homogenous network similar to that occupied by NMY-2 (Fig. S6A; Munro et al., 2004). PAR-1(T983A) was also present on the posterior cortex, but in a more dispersed distribution. Enrichment of PAR-1(T983A)::GFP at the anterior cortex was eliminated by *nmy-2* (RNAi) (Fig. S6A). We constructed a strain co-expressing NMY-2::mKade2 and PAR-1::GFP or PAR-1(T983A)::GFP. NMY-2 and PAR-1 did not colocalize throughout the cortex, but we could detect enrichment of PAR-1 in the brightest NMY-2 clusters on the anterior cortex (Fig. S6B). Pearson correlation coefficient measurements confirmed that PAR-1 and NMY-2 partially colocalize (Fig. S6C). We conclude that NMY-2 and PAR-1 co-exist in certain regions of the cortex and that NMY-2 affects the distribution of PAR-1, which is consistent with a potential role for NMY-2 in regulating PAR-1 activity.

## DISCUSSION

### Parallel regulation of PAR-1 by PKC-3 and the KA1 domain

**Fig. 8.**
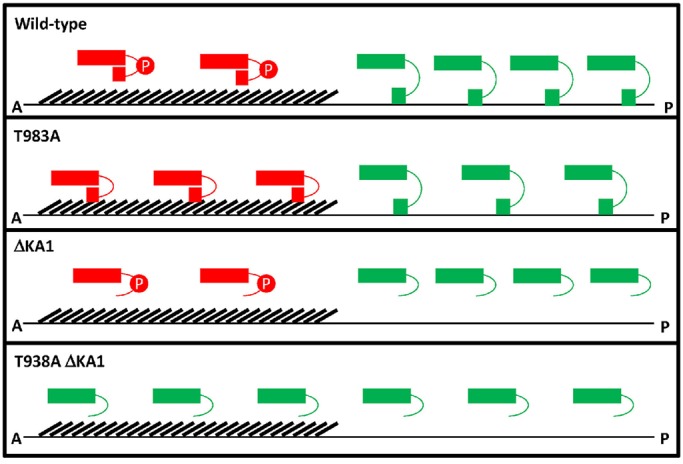
**Working model for PAR-1 regulation.** PAR-1 kinase domain (rectangle) is either active (green) or inactivated (red) by phosphorylation by PKC-3 (red P) and/or by the KA1 domain (square). Binding to membranes (thin lines) relieves KA1-dependent auto-inhibition. The domain that is enriched in NMY-2 (hashes) prevents PAR-1 from accessing membranes in anterior. Membranes are plasma membrane and membrane network of the endoplasmic reticulum. Wild type: In the anterior, PAR-1 kinase activity is inhibited by PKC-3. PKC-3 also increases PAR-1 mobility in the anterior, which causes modest enrichment in the posterior. In the posterior, auto-inhibition of PAR-1 is lifted by binding of the KA1 domain to membranes. PAR-1(T983A): In the anterior, auto-inhibited PAR-1 binds to a cortical factor, which prevents access to membranes. In the posterior, auto-inhibition of PAR-1(T983A) is lifted by binding of the KA1 domain to membranes. PAR-1(ΔKA1): In the anterior, PAR-1 kinase activity is inhibited by PKC-3. PKC-3 also increases PAR-1 mobility in the anterior causing modest enrichment in the posterior. In the posterior, PAR-1 is active (no auto-inhibition). PAR-1(T983A ΔKA1): In the anterior and posterior, PAR-1 is active (no auto-inhibition and no inhibition by PKC-3).

Using genome editing, we have generated *par-1* mutants that lack regulation by PKC-3, the KA1 domain, or both. We find that either mode of regulation is sufficient to polarize PAR-1 activity in zygotes. In the absence of regulation by PKC-3, PAR-1 was no longer excluded from the anterior, but MEX-6 and P granules still polarized. In the absence of the KA1 domain, PAR-1 no longer localized to cortical membranes, but formed a shallow gradient in the posterior cytoplasm and MEX-6 and P granules still polarized. Our findings are consistent with previous studies that have reported asymmetric P granules in embryos with mislocalized PAR-1 (Cheeks et al., 2004) or cytoplasmic-only PAR-1 (Boyd et al., 1996; Labbé et al., 2006). Mutating both the PKC-3 site and the KA1 domain, however, resulted in upregulation of PAR-1 activity throughout the zygote and symmetric P granules. We conclude that inhibition by PKC-3 and by the KA1 domain function in parallel to restrict PAR-1 activity to the posterior (Fig. 8).

### Phosphorylation of T983 by PKC-3 creates a posterior-rich PAR-1 activity gradient

Consistent with observations in other systems (Doerflinger et al., 2010; Suzuki et al., 2004), substituting an alanine at the PKC-3 phosphorylation site T983 eliminated enrichment of PAR-1 in the posterior domain. Protein gradients can arise in the cytoplasm because of local changes in protein mobility that are triggered by reversible phosphorylation (Griffin et al., 2011). Similarly, PKC-3 could generate the PAR-1 gradient by increasing PAR-1 mobility in the anterior cytoplasm, perhaps by preventing binding to a sub-diffusive anchor that is present throughout the cytoplasm (Lipkow and Odde, 2008). Phosphorylation by PKC-3 interferes with PAR-1 binding to lipids *in vitro* (Ramanujam et al., 2018), so one possibility is that PAR-1 interacts with membranes of the endoplasmic reticulum (ER) in the cytoplasm. The PAR-1 cytoplasmic gradient is only weakly affected by deletion of the KA1 domain in zygotes, but becomes progressively more disrupted with each P cell division. One possibility is that, in the absence of the KA1 domain, PAR-1 basal mobility is increased overall, and this interferes with regulation by PKC-3, especially as cell size decreases in the P lineage. Phosphorylation by PKC-3 has been shown to interfere with PAR-1 binding to lipids *in vitro* (Ramanujam et al., 2018). The membranous network of the ER occupies the entire cytoplasmic space of the *C. elegans* embryo (Poteryaev et al., 2005). An intriguing possibility is that C-terminal sequences, including the KA1 domain, allow PAR-1 to interact dynamically with ER membrane lipids, or another ubiquitous anchor, to slow down PAR-1 diffusion and coincidently activate kinase activity.

Although PAR-1 protein asymmetry is a visible output of regulation by PKC-3, modeling studies have shown that the PAR-1 protein gradient is too shallow to generate the steeper MEX-5/6 gradient (Griffin et al., 2011). In tissue culture cells, phosphorylation by aPKC was shown to reduce the kinase activity of human Par1b (Hurov et al., 2004). Therefore, we suggest that, in addition to generating a weak PAR-1 protein gradient, phosphorylation by PKC-3 also generates a PAR-1 kinase activity gradient. How phosphorylation at T983, a residue outside of the kinase domain, affects PAR-1 kinase activity is not known. This inhibition is unlikely to require auto-inhibition by the KA1 domain as regulation by T983 is still active when the KA1 domain is deleted (see below).

*par-1(T983A)* zygotes polarized MEX-5/6 and P granules only weakly during polarity establishment and did not maintain these asymmetries during mitosis. In the wild type, the AB blastomere divides ∼2 min before the P_1_ blastomere, and AB and P_1_ descendants continue to divide asynchronously (Tavernier et al., 2015). In contrast, in *par-1(T983A)* embryos, AB, P_1_, and their descendants divided in synchrony. Cell cycle asynchrony has been correlated with asymmetric enrichment in the two-cell stage of the cell cycle regulators PLK-1, CDC-25 and Cyclin B (Tavernier et al., 2015). Best understood is PLK-1, which binds to MEX-5 and requires *mex-5* and *mex-6* activities to segregate preferentially to the AB cell (Budirahardja and Gonczy, 2008; Nishi et al., 2008). We suggest that the weak polarization activity of *par-1(T983A)*, especially during mitosis, is not sufficient to support asymmetric segregation of PLK-1 and other cell cycle regulators. Our findings confirm that, as in other systems, regulation by aPKC/PKC-3 is essential for robust cell polarization by PAR-1.

### Auto-inhibition by the KA1 domain creates a posterior-rich PAR-1 activity gradient in a NMY-2 dependent manner

Three lines of evidence indicate that auto-inhibition by the KA1 domain also generates a PAR-1 activity gradient. First, as first shown for human MARK1 (Emptage et al., 2017, 2018), the KA1 domain of *C. elegans* PAR-1 inhibits kinase activity *in vitro*. This inhibition requires K1170 and R1171, the same basic residues that are required for auto-inhibition in MARK1 (Emptage et al., 2017). Second, deleting or mutating the KA1 domain has a small but detectable impact on the PAR-1 activity gradient in zygotes, causing an expansion of PAR-1 activity towards the anterior. Third, the KA1 domain is essential for the polarized activity of PAR-1(T983A). Combining the T983A mutation with mutations in K1170 and R1171 (or a deletion in the KA1 domain) resulted in dramatic upregulation of PAR-1 activity throughout the zygote and dominant maternal effect sterility (also see below). These observations suggest that auto-inhibition by the KA1 domain tunes PAR-1 activity to create a steep PAR-1 activity gradient across the zygote.

We consider three possibilities for how this activity gradient might be generated. As with mutations in the KA1 domain, loss of the non-muscle myosin NMY-2 results in upregulation of PAR-1 activity throughout the cytoplasm of *par-1(T983A)* zygotes. The C-terminus of PAR-1 binds to NMY-2 (Guo and Kemphues, 1996) and PAR-1(T983A) partially colocalizes with NMY-2 in the anterior cortex in a pattern that is dependent on NMY-2. An intriguing possibility is that binding to NMY-2 in the anterior prevents PAR-1 from accessing membrane phospholipids that would otherwise relieve auto-inhibition. In this way, NMY-2 could directly influence PAR-1 activity by locking PAR-1 in the auto-inhibited form. A second possibility is that cortical flows that are generated by NMY-2 localize another factor that regulates auto-inhibition by the KA1 domain. PAR-2 is a membrane-associated protein in the posterior cortex, but PAR-2 is not essential to polarize PAR-1 activity (Boyd et al., 1996; Labbé et al., 2006; Ramanujam et al., 2018). Interestingly, PAR-2 becomes essential to polarize zygotes when cortical flows are inhibited and myosin remains uniformly distributed, which suggests that PAR-2 could play a non-essential role in helping PAR-1 overcome inhibition by myosin (Motegi et al., 2011). A third possibility is that regulation by the KA1 domain is inherently dynamic and reduces PAR-1 activity evenly throughout the cytoplasm. In this model, other factors that function in parallel with PAR-1 to polarize the zygote would need to be spatially regulated (Gallo et al., 2010; Liro et al., 2018). For example, the activity of the PP2A phosphatase complex that reverses phosphorylation of MEX-5/6 by PAR-1 could be weakly polarized and require a low basal level of PAR-1 activity (dependent on KA1) to polarize MEX-5/6 effectively (Griffin et al., 2011). We do not favor this model because there is no evidence at this time for asymmetric localization of PP2A, and because PAR-1(T983) also patterns the distribution of PAR-3, a protein that is not known to require PP2A for polarization. Although the exact mechanism remains to be determined, our results indicate that the KA1 domain functions as an auto-inhibitory domain *in vivo* that, in collaboration with NMY-2, tunes PAR-1 activity in space to create a net activity gradient.

A connection between PAR-1, myosin and the actin cytoskeleton has also been observed in other organisms. For example in *Drosophila*, PAR-1 regulates myosin activity during border cell migration (Majumder et al., 2012) and PAR-1 localization to the posterior cortex of the oocyte requires the actin cytoskeleton (Doerflinger et al., 2006). In sea urchins, PAR-1 localizes with aPKC on the apical cortex of embryonic blastomeres, and localization of the PAR complex to apical cortices depends on myosin (Ossipova et al., 2007), as observed here for PAR-1(T983A). We suggest that modular regulation of PAR-1 activity by aPKC and by membrane-associated proteins that enhance or relieve auto-inhibition allows different cell types to restrict PAR-1 activity to different cellular locations.

### After the zygote stage, the KA1 domain is essential for asymmetric localization of PAR-1 and for fertility

Deletion of the KA1 domain had only a weak effect on PAR-1 asymmetry in zygotes, but strongly reduced asymmetric enrichment of PAR-1 and P granules in subsequent divisions, and *par-1(ΔKA1)* embryos developed into sterile adults. This maternal-effect sterile phenotype contrasts with that of embryos that lack all *par-1* activity, or that express symmetric *par-1(T983A)*, which do not survive embryogenesis (maternal-effect lethality). Maternal-effect sterility was also observed among rare survivors of a hypomorphic allele of *par-1* (Guo and Kemphues, 1995). These observations suggest that, after the zygote stage, asymmetric segregation of PAR-1 is no longer essential to specify the fates of somatic blastomeres, and is required exclusively for germline development. Interestingly, one copy of *par-1(T983A ΔKA1)* or *par-1(T983A KKSS)* resulted in almost fully penetrant dominant maternal-effect sterility, indicating that one dose of deregulated constitutively active PAR-1 is also detrimental to fertility. One possibility is that polarized PAR-1 activity in the P lineage is essential to segregate crucial fertility factors to the nascent germline. The identity of these crucial fertility factors is not known. P granules are unlikely candidates, as embryonic P granules are not essential for fertility (Gallo et al., 2010), but other germ plasm factors could be involved. Alternatively, PAR-1 itself could be required for the development of the nascent germline. Asymmetric segregation of PAR-1 enriches PAR-1 in the germline founder cell P_4_ and its daughters, the primordial germ cells Z2 and Z3. Z2 and Z3 form asymmetric lobes in mid-embryogenesis that could potentially require PAR-1 activity (Abdu et al., 2016). A role for *C. elegans* PAR-1 in fertility is reminiscent of the role of *Drosophila* PAR-1 in organizing germ cell determinants (Shulman et al., 2000), and suggests the existence of an ancient link between PAR-1 kinases and specification of the embryonic germline.

## MATERIALS AND METHODS

### Strains and alleles

*C. elegans* were cultured according to standard methods (Brenner, 1974). All strains (Table S2) were maintained at 20°C.

### Plasmid construction

Table S3 lists all plasmids used in this study. A Q5 site-directed mutagenesis kit (New England Biolabs) was used to integrate the 6xHis coding sequence at the N-terminus of the maltose-binding protein (MBP) coding sequence in vector pMAL-C5E (New England Biolabs), to generate pAF9. The 6xHis::MBP::PAR-1 and 6xHis::MBP::PAR-1(ΔKA1) expression vectors were constructed by cloning *par-1* and *par-1(ΔKA1)* open reading frames (ORFs) into pAF9. The 6xHis::MBP::MEX-5(452-460) expression vector was constructed by cloning *mex-5* ORFs into pAF9 using a Gibson assembly cloning kit (New England Biolabs). The 6xHis::KA1 expression vector (pAF10) was constructed by cloning *par-1(1089-1192)* ORF into pET28a (Novagen) using a Gibson assembly cloning kit (New England Biolabs). A Q5 site-directed mutagenesis kit (New England Biolabs) was used to generate the 6xHis::KA1(KRSS) expression vector (pAF11) (Tables S3,S4).

### RNA interference

RNAi knockdown experiments were performed by feeding on HT115 bacteria (Timmons and Fire, 1998) or by injection of double-strand RNA (dsRNA) (*par-1* and *nmy-2*) (Timmons and Fire, 1998). Feeding constructs for F58B6.3 (*par-2*), W02A2.7 (*mex-5*), and AHG.5 (*mex-6*) were obtained from the Ahringer or OpenBiosystem libraries. The empty pL4440 vector was used as negative control. RNAi bacteria were grown at 37°C in lysogeny broth (LB)+ampicillin (100 µg/ml) media for 5-6 h, induced with 5 mM isopropyl β-D-1-thiogalactopyranoside (IPTG) for 30 min, plated on nematode nutritional growth media (NNGM)+ampicillin (100 µg/ml)+IPTG (1 mM) plates, and grown overnight at room temperature. L4 hermaphrodites were allowed to feed for 24 h at 20°C before examination.

For *par-1* and *nmy-2* RNAi experiments, T7 primers (Table S4) were used to amplify a gene specific fragment from genomic DNA. dsRNA was produced using Ribomax T7 *in vitro* transcription system (Promega) and the RNA was purified using RNeasy (Qiagen). dsRNA was microinjected into both distal gonad arms of young adult hermaphrodites. The injected worms were recovered using standard procedures (Paix et al., 2015) and incubated at 20°C for 24-30 h before examination.

### CRISPR-mediated genome editing

Genome editing was performed using CRISPR/Cas9 as described in Paix et al. (2015). For all edits (except PAR-1::GFP), in addition to the desired mutation, we also introduced silent mutations to generate novel restriction sites to facilitate screening (see Tables S2 and S4 for details).

### Confocal microscopy

Fluorescence microscopy was performed using a Zeiss Axio Imager with a Yokogawa spinning-disc confocal scanner. Images were taken using Slidebook v6.0 software (Intelligent Imaging Innovations) using either a 63× or 40× objective. Equally normalized images were exported by Slidebook v6.0, and contrasts of images were equally adjusted between control and experimental sets using ImageJ. For live imaging, embryos were dissected from adult hermaphrodites in M9 solution, mounted onto 2% agarose pads and imaged at 20°C. We noticed that *par-1(T983A)* embryos were sensitive to compression under the glass coverslip, which gave variable results. To eliminate this variability, we only examined embryos that were compressed by the coverslip during or after the pronuclear migration stage (cell fate determinants within these zygotes are polarized while inside the hermaphrodite uterus).

### Image quantification

Quantification of GFP fluorescence using line scan analysis: Equally normalized images of PAR-1::GFP, MEG-3::GFP, MEX-6::GFP and mCherry::MEX-5 of indicated genotypes were quantified using ImageJ, with the ‘Plot Profile’ functionality. To allow for an equal number of measurements between embryos of slightly varied length, each individual zygote was cropped using ImageJ and sized to be an absolute length of 230 pixels using Adobe Illustrator. The absolute pixel number height of each zygote image remained unchanged in this manipulation. Fluorescence intensity was averaged along a 50 pixel-wide, 230 pixel-long line that spanned the length of each zygote (0% anterior most measurement, 100% posterior-most measurement) using the ‘Plot Profile’ function of ImageJ.

Quantification of PAR-3 cortical clusters: Cortical PAR-3::GFP intensity values of the cortical plane of indicated genotypes were normalized to the maximum embryo intensity. The area of the posterior domains was quantified using the ‘Area’ measurement of ImageJ. An intensity threshold was set and the absolute number of PAR-3 clusters was manually counted in the posterior domain.

Pearson correlation coefficients were calculated for a square region of interest (ROI) within the ­anterior domain of non-thresholded GFP::PAR-1 and NMY-2::mKade2 images using the ‘Coloc 2’ application within ImageJ. For statistical analysis, the samples were compared using an unpaired two-sided Student's *t*-test (GraphPad Prism).

### Purification of 6xHis::MBP::PAR-1 variants

Recombinant 6xHis::MBP::PAR-1 and 6xHis::MBP::PAR-1(ΔKA1) were expressed in *Escherichia coli* Rosetta (DE3) cells (EMD Millipore). 6xHis::MBP::PAR-1 and 6xHis::MBP::PAR-1(ΔKA1) were purified as follows. Induced cells were spun at 6000 ***g*** for 15 min and the pellet was resuspended in Buffer A [20 mM HEPES (pH 7.4), 200 mM NaCl, 1 mM EDTA, 1 mM EGTA, 1 mM DTT, Roche complete EDTA-free protease inhibitor tablet], lysed by sonication and spun at 25,000 ***g*** for 15 min. The supernatant was passed over an MBPTrap column (GE Healthcare) and washed with Buffer B [20 mM HEPES (pH 7.4), 200 mM NaCl, 1 mM DTT]. The column was equilibrated with Buffer C [20 mM HEPES (pH 7.6), 100 mM NaCl] and eluted with Buffer D [20 mM HEPES (pH7.6), 100 mM NaCl, 10 mM maltose]. The supernatant was passed over a HiTrap SP column (GE Healthcare). The column was washed with Buffer C and eluted using a linear salt gradient with Buffer E [20 mM HEPES (pH 7.6), 1.5 M NaCl]. Fractions that contained PAR-1 were pooled and fractionated on an S300 column (GE Healthcare) using Buffer F [20 mM HEPES (pH7.6), 200 mM NaCl, 20% w/v glycerol].

### Purification of 6xHis::KA1 variants

Recombinant 6xHis::KA1 and 6xHis::KA1(KRSS) were expressed in *E. coli* Rosetta (DE3) cells. 6xHis::KA1 and 6xHis::KA1(KRSS) were purified as follows. Induced cells were spun at 6000 ***g*** for 15 min and the pellet was resuspended in Buffer A [20 mM HEPES (pH 8.0), 200 mM NaCl, 20 mM imidazole, 1 mM TCEP, Roche complete EDTA-free protease inhibitor tablet], lysed by sonication and spun at 25,000 ***g*** for 15 min. The supernatant was passed over a HisTrap column (GE Healthcare), washed with Buffer A and eluted with Buffer B [20 mM HEPES (pH 7.6), 200 mM NaCl, 250 mM imidazole]. The eluent was diluted 1:1 with Buffer C [20 mM HEPES (pH 7.6), 100 mM NaCl] and passed over a HiTrap SP column. The column was washed with Buffer C and eluted using a linear salt gradient with Buffer D [20 mM HEPES (pH 7.6), 1.5 M NaCl]. Fractions that contained 6xHis::KA1 were pooled and fractionated on a S200 column (GE Healthcare) using Buffer E [20 mM HEPES (pH 7.6), 200 mM NaCl, 20% w/v glycerol].

### Purification of 6xHis::MBP::MEX-5(445-468)

Recombinant 6xHis::MBP::MEX-5 was expressed in *E. coli* Rosetta (DE3) cells. Induced cells were spun at 6000 ***g*** for 15 min and the pellet was resuspended in Buffer A [20 mM HEPES (pH 8.0), 200 mM NaCl, 20 mM imidazole, 1 mM TCEP, Roche complete EDTA-free protease inhibitor tablet], lysed by sonication and spun at 25,000 ***g*** for 15 min. The supernatant was passed over a HisTrap column, washed with Buffer B [20 mM HEPES (pH 8.0), 500 mM NaCl, 20 mM imidazole, 0.2% w/v Triton-X] and eluted with Buffer C [20 mM HEPES (pH 7.6), 200 mM NaCl, 250 mM imidazole]. Fractions that contained 6xHis::MBP::MEX-5(452-460) were pooled and fractionated on a S200 column using Buffer D [20 mM HEPES (pH7.6), 200 mM NaCl, 20% w/v glycerol].

### Kinase assay

Time course kinase reactions were performed with 40 nM MBP::PAR-1 or MBP::PAR-1(ΔKA1) at 30°C in the presence of P^32^-ATP for indicated times in a kinase reaction buffer [20 mM HEPES (pH 7.4), 8 µM MgCl_2_, 4 µM ATP 1 mg/ml BSA, 15% w/v glycerol] with 1 µM MBP::MEX-5(445-468). All reactions were stopped by the addition of 4× Laemmli SDS buffer. Proteins were resolved using SDS PAGE on two 7% Tris-Acetate gels (Thermo Fisher Scientific). One gel was stained with Coomassie and an image was taken with an iPhone (Apple). Phosphorylation of MEX-5 was visualized using autoradiography of the second unstained gel. KA1 trans-inhibition reactions were performed with 400 nM MBP::PAR-1(ΔKA1) at 30°C for 10 min in a kinase reaction buffer [20 mM HEPES (pH 7.4), 8 µM MgCl_2_, 4 µM ATP, 1 mg/ml BSA, 15% w/v glycerol] and 5 µM MBP::MEX-5(445-468). Titration of recombinant KA1 domain protein was carried out with His(6)::KA1 or His(6)::KA1(KRSS) at 0, 5 mM, 20 mM and 50 mM concentrations. Reactions were performed at 30°C in the presence of P^32^-ATP for 10 min. All reactions were stopped by the addition of 4× Laemmli SDS buffer. Proteins were resolved using SDS PAGE on a 7% Tris-Acetate gel (Thermo Fisher Scientific). The gel was stained with Coomassie and an image was taken with an iPhone (Apple). Phosphorylation of MEX-5 was visualized by autoradiography of the stained gel.

## Supplementary Material

Supplementary information
