# Supplementary material for: Spatial regulation of the polarity kinase PAR-1 by parallel inhibitory mechanisms
Source: Development. 2019 Mar 25;146(6):dev171116. doi: 10.1242/dev.171116 (PMC6451319; doi:10.1242/dev.171116)
Supplement: Supplementary information [file develop-146-171116-s1.pdf]

Fig. S1

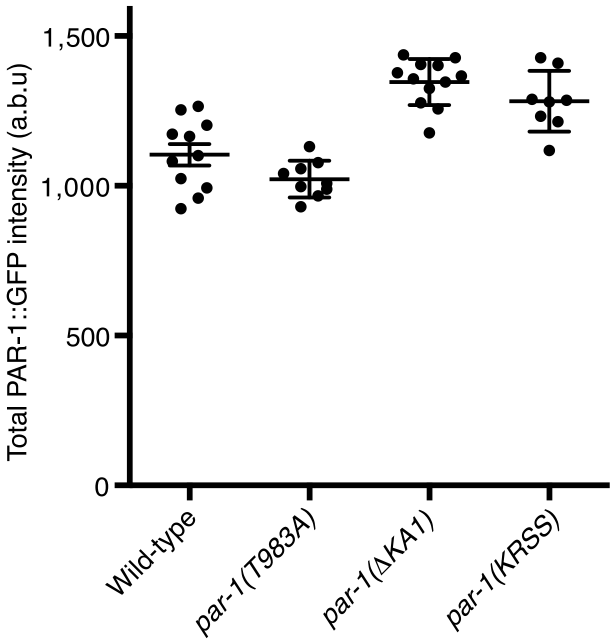

Fig. S1: PAR-1 alleles are expressed at comparable levels.

Graph showing the average PAR-1::GFP intensity in zygotes of the indicated genotypes. Each dot corresponds to one zygote; at least 8 zygotes at pronuclear meeting were analyzed for each genotype. The entire volume of each live zygote was captured as a Z-stack via confocal microscopy to generate a sum projection. Average PAR-1::GFP intensity was determined for each zygote. Error bars represent the standard error of the mean (SEM).

Development • Supplementary information

Fig. S2

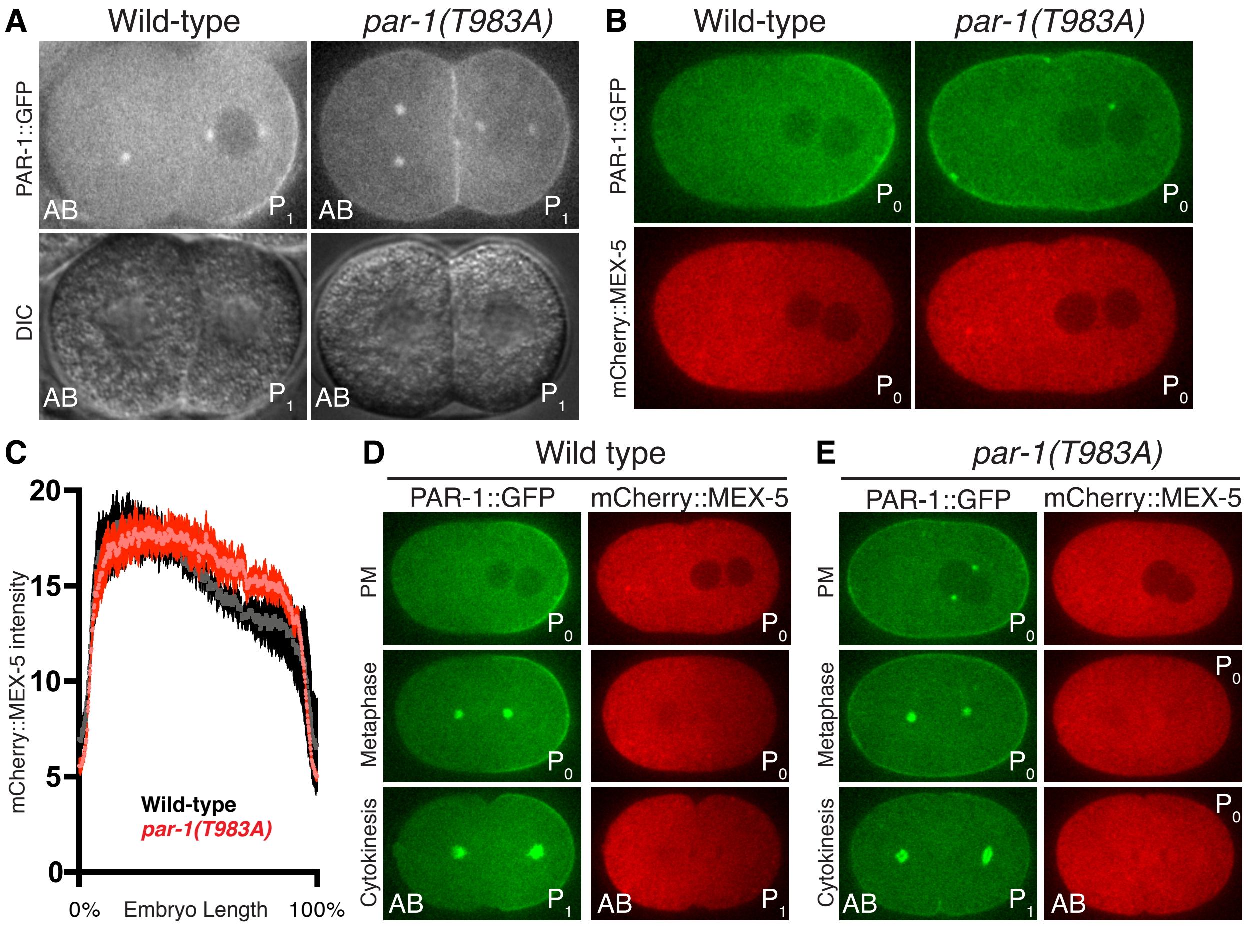

Fig. S2: Characterization of *par-1(T983A)*.

(A) Photomicrographs of live 2-cell embryos of the indicated genotypes expressing PAR-1::GFP (fluorescence top panels, and Nomarski, bottom panels). Unlike in wild-type, in *par-1(T983A)*, AB and P_1 are preparing to divide synchronously as evidenced by lack of GFP exclusion from nuclei in both AB and P_1 .
(B) Photomicrographs of live wild-type and *par-1(T983A)* zygotes co-expressing PAR-1::GFP and mCherry::MEX-5.
(C) Graphs plotting the relative signal intensity of the mCherry::MEX-5 in zygotes of the indicated genotypes. 5 zygotes at pronuclear meeting were analyzed for each condition. Error bars represent the standard error of the mean (SEM). The MEX-5 gradient in *par-1(T983A)* is shallower than in wild-type.
(D-E) Photomicrographs of live zygotes of indicated genotypes expressing PAR-1::GFP and mCherry::MEX-5 staged at (from top to bottom) pronuclear meeting (PM), metaphase, and cytokinesis. The MEX-5 gradient is not maintained during mitosis in *par-1(T983A)*.

Development • Supplementary information

Fig. S3

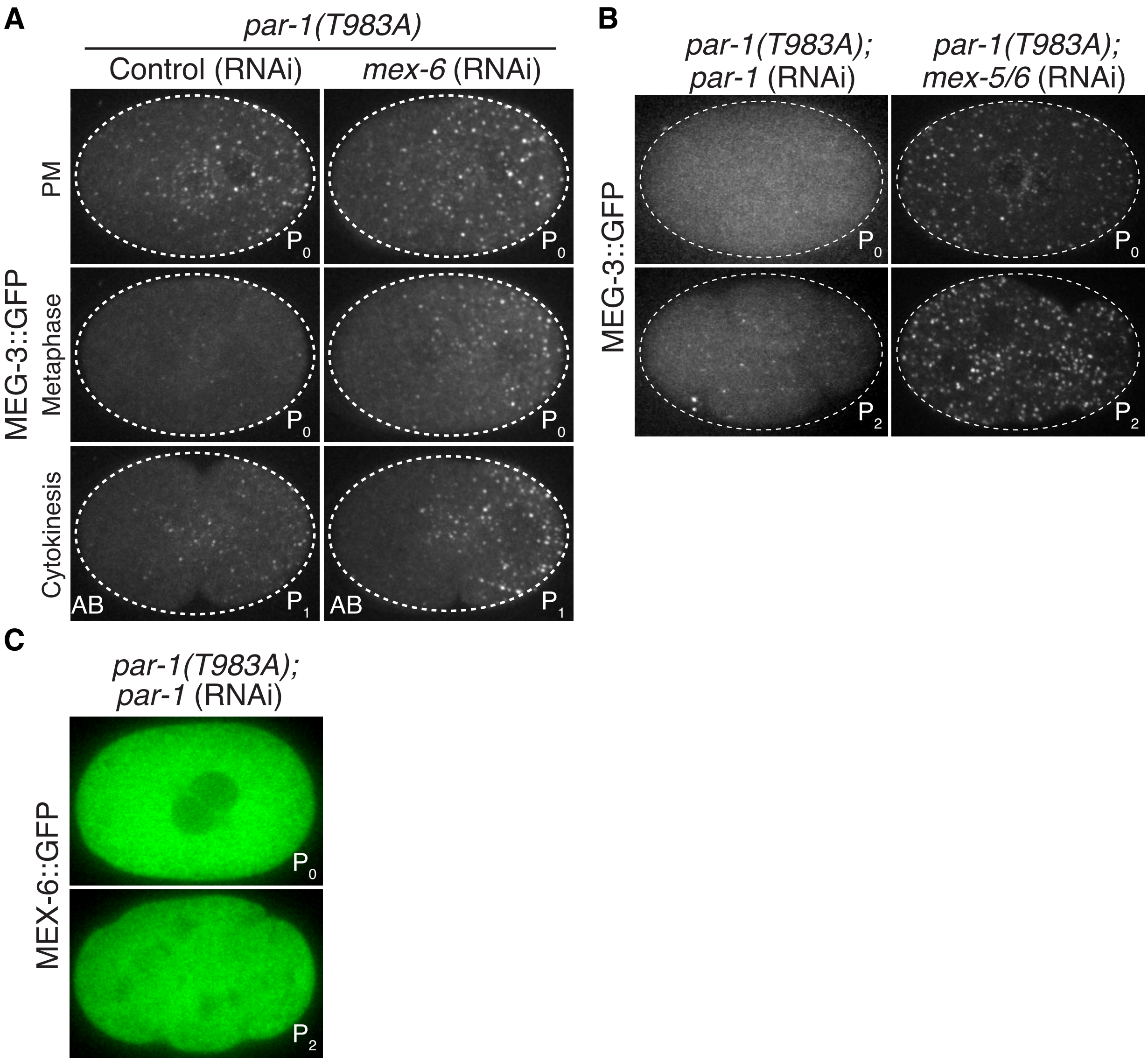

Fig. S3: Analysis of *par-1(T983A)* cell fate patterning activity.

(A) Photomicrographs of live MEG-3::GFP in *par-1(T983A)*, and *par-1(T983A);mex-6(RNAi)* zygotes staged at (from top to bottom) pronuclear meeting (PM), metaphase, and cytokinesis. P granules dissolve in metaphase in *par-1(T983A)* zygote and this phenotype is rescued by *mex-6* (RNAi).
(B-C) Photomicrographs of live *par-1(T983A)* zygotes with GFP::MEG-3 **(B)** or GFP::MEX-6 **(C)** depleted for *par-1* or *mex-5/6* by RNAi. Depletion of *par-1* and *mex-5/6* eliminates MEX-6 and P granule asymmetry (Gallo et al., 2010; Schubert et al., 2000).

Fig. S4

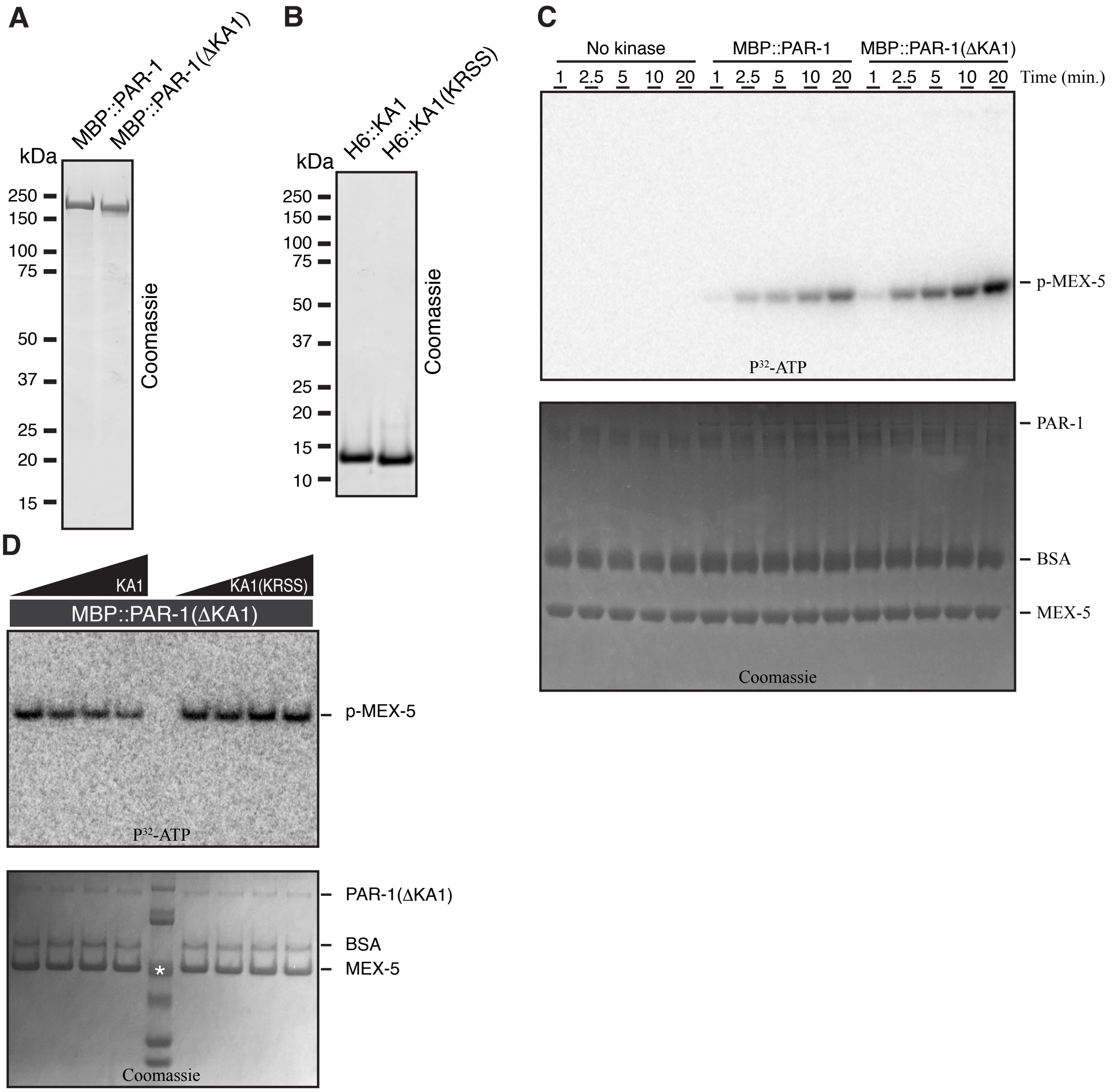

Fig. S4: *in vitro* analysis of PAR-1 kinase activity.

(A-B) Coomassie stained SDS-PAGE page gels of indicated recombinant proteins. **(C-D)** Shown are full gels of the data presented in Fig. 4. **(C)** Autoradiograph of a time course kinase assay using recombinant MBP::PAR-1 or MBP::PAR-1(ΔKA1) kinase, and MBP::MEX-5(452-460) as a substrate. Reactions were performed in the presence of P^{32}-ATP for indicated times. Bottom panel shows a Coomassie blue stained gel to control for loading of PAR-1 and MEX-5. **(D)** Autoradiograph of a kinase assay using recombinant MBP::PAR-1(ΔKA1) kinase and titrating in His(6)::KA1 or His(6)::KA1(KRSS) at 0, 5mM, 20mM, 50mM concentrations. Reactions were performed in the presence of P^{32}-ATP for 10 minutes. Bottom panel shows Coomassie blue staining to control for loading of PAR-1 and MEX-5. *Indicates molecular mass protein standard marker.

Fig. S5

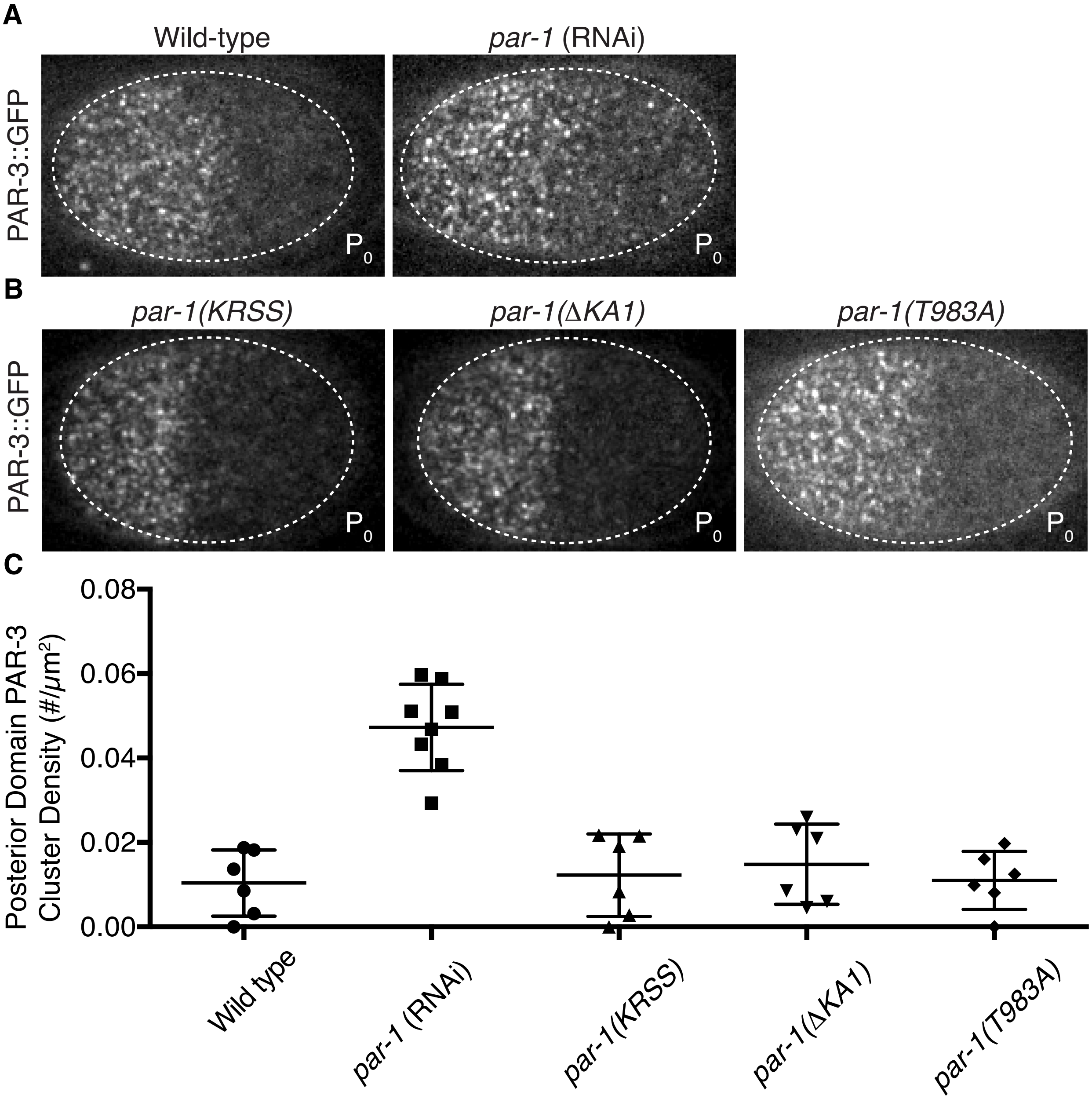

Fig. S5: Cortical enrichment of PAR-1 is not required to pattern PAR-3.

(A) Photomicrographs of live wild-type zygotes with GFP-tagged PAR-3. Depletion of *par-1* by RNAi increases the levels of PAR-3::GFP in the posterior cortex as reported by (Sailer et al., 2015). **(B)** Photomicrographs of live *par-1(KRSS)*, *par-1(ΔKA1)*, or *par-1(T833A)* zygotes with GFP-tagged PAR-3. All three *par-1* mutants are indistinguishable from wild-type. **(C)** Graph plotting the density of PAR-3 clusters in the posterior domain of the indicated genotypes. At least 6 zygotes staged at late metaphase were analyzed for each genotype. Error bars represent the standard error of the mean (SEM).

Development • Supplementary information

Fig. S6

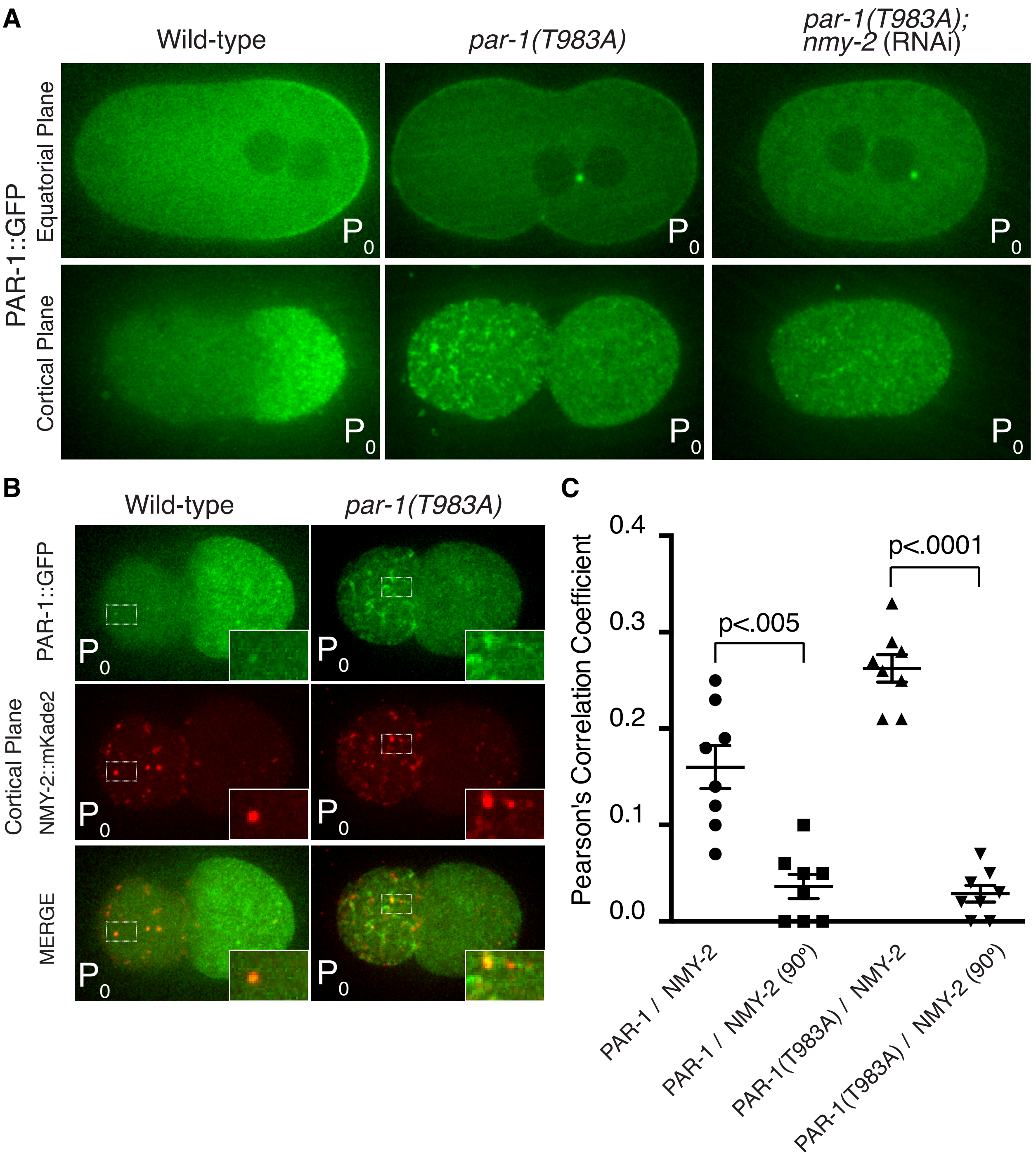

Development • Supplementary information

Fig. S6: *PAR-1(T983A)* is enriched on the anterior cortex.

(A) Photomicrographs of PAR-1::GFP in zygotes of the indicated genotypes. Top row shows equatorial plane, bottom row shows cortical plane of the same embryo to show distribution of PAR-1::GFP in the cortex. Wild-type PAR-1::GFP enriches in the posterior cortex due to exclusion by PKC-3 in the anterior cortex. PAR-1::GFP(T983A), in contrast, shows transient enrichment in the anterior cortex during zygote polarization. Depletion of NMY-2 eliminates this asymmetry.
(B) Photomicrographs of live zygotes of indicated genotypes co-expressing PAR-1::GFP and NMY-2::mKade2. Insets show 2.6-fold magnification of the regions shown in the dash-lined boxes.
(C) Pearson's correlation coefficients (PCC) were calculated for a square region of interest (ROI) within the anterior domain of PAR-1::GFP and NMY-2::mKade2 images. As a negative control, the PCC was also calculated for the same cognate ROI, but after rotation of NMY-2::mKade2 ROI by 90 degrees. 8 zygotes at pronuclear meeting were analyzed for each condition. Error bars represent the standard error of the mean (SEM).

Development • Supplementary information

Table S1: Maternal effect embryonic lethality and sterility of *par-1* mutant strains

Click here to Download Table S1

Table S2: Strains utilized in the study

Click here to Download Table S2

Table S3: Plasmids utilized in the study

Click here to Download Table S3

Table S4: DNA and RNA reagents utilized in the study

Click here to Download Table S4

Development • Supplementary information
